# Validation of a new tool for evaluating subjects’ satisfaction with medicine package leaflets: a cross-sectional descriptive study

**DOI:** 10.1590/1516-3180.2019.0123160919

**Published:** 2020-01-13

**Authors:** Carla Pires, Pedro Joel Rosa, Marina Vigário, Afonso Cavaco

**Affiliations:** I PhD. Pharmacist and Invited Professor, Department of Pharmacotherapy, Research Center for Biosciences and Health Technologies (CBIOS), Universidade Lusófona, Campo Grande, Lisbon, Portugal.; II PhD. Psychologist, Statistician and Assistant Professor, Psychology Department, School of Psychology and Life Sciences, Lusophone University of Humanities and Technologies (ULHT), Lisbon, Portugal; Human Environment Interaction Lab (HEI-lab), ULHT, Lisbon, Portugal; Instituto Universitário de Lisboa (ISCTE-IUL), Cis-IUL, Lisbon, Portugal.; III PhD. Linguist and Associate Professor, Department of General and Romance Linguistics, School of Arts and Humanities & Centre of Linguistics, University of Lisbon, Lisbon, Portugal.; IV PhD. Pharmacist and Associate Professor, Department of Social Pharmacy, School of Pharmacy, Research Institute for Medicines and Pharmaceutical Sciences, University of Lisbon, Lisbon, Portugal.

**Keywords:** Product labelling, Psychometrics, Legislation, pharmacy, Pharmaceutical preparations, Patient satisfaction, Package leaflets, Package inserts, Medicines, Validation, Likert scale, Regulation

## Abstract

**BACKGROUND:**

Package leaflets of medicines need to be intelligible, but tools for their evaluation are scarce.

**OBJECTIVE:**

To validate a new tool for assessing subjects’ satisfaction with medicine package leaflets (LiS-RPL).

**DESIGN AND SETTING:**

Cross-sectional descriptive study conducted in two regions of Portugal (Lisbon and Centre).

**METHODS:**

503 participants (53.1% male) were selected according to convenience and homogenously distributed into three groups: 1 to 6; 7 to 12; and > 12 years of schooling. LiS-RPL was developed based on international regulation guidelines and was initially composed of 14 items. Twelve package leaflets were tested. Dimensionality calculations included: exploratory factor analysis and minimum rank factor analysis; Kaiser-Meyer-Olkin index and Bartlett’s sphericity test to assess matrix adequacy for exploratory factor analysis; exploratory bifactor analysis with Schmid-Leiman solution to detect possible existence of a broad second-order factor; and Bentler’s Simplicity Index and Loading Simplicity Index to assess factor simplicity. Diverse coefficients were calculated to assess reliability.

**RESULTS:**

Minimum rank factor analysis detected a two-factor or single-factor structure. Exploratory factor analysis with 12 items showed a two-factor structure, explaining 69.11% of the variance. These items were strongly correlated with each other (r = 0.80). Schmid-Leiman: all items seemed to represent the general factor (loadings above 0.50), which was 76.4% of the extracted variance. Simplicity indices were good (percentile 99): Bentler’s Simplicity Index of 0.99 and Loading Simplicity Index of 0.48. Internal consistency indexes indicated good reliability. LiS-RPL was shown to be homogenous.

**CONCLUSION:**

LiS-RPL is a validated tool for evaluating subjects’ satisfaction with medicine package leaflets.

## INTRODUCTION

According to the European Guideline on the Readability of the Labelling and Package Leaflet of Medicinal Products for Human Use, package leaflets of medicines (from here on, referred to simply as package leaflets) need to be simple, clear and comprehensible. This is why legibility tests involving groups of patients need to be conducted, so as to ensure that package leaflets are readable.^1^ Additionally, in designing package leaflets, special focus should be given to elements such as use of a simple writing style, adequate font size and layout (e.g. line spacing, use of bullet points and consistent headings) and adequate contrast.^2^

Questionnaires using Likert scales (i.e. a psychometric scale for questionnaires comprising an intermediate value and an odd number of alternatives, usually five or seven) are commonly used in readability/legibility tests to evaluate the readability of package leaflets.^3^^,^^4^^,^^5^^,^^6^ In these tests, the scores of the Likert scales are often used to check readability after package leaflets have been optimized, i.e. test-retest methodology is usually applied.^3^^,^^4^^,^^6^

These scales are easy to apply and often present high response rates (84-91%) and completion rates (70-85%).^4^^,^^7^ Another advantage is that 20-item questionnaires in which responses are assessed using a Likert scale can be completed fast: in less than five minutes if the questionnaire is simple. Lastly, these tools give rise to good internal consistency, reliability and construct validity.^7^

Although to our knowledge no Likert scale-based tool has been specifically developed by the European Medicine Agency to specifically evaluate package leaflets in a given language,^1^^-^^2^ some studies have reported on this type of tool for other materials in general. For instance, DISCERN, which includes 16 items, and EQUIP, which contains 20 items, can be applied in English, German or Portuguese. DISCERN and EQUIP are used to measure the perceived quality of written health-related information, including its graphical presentation (for EQUIP), as seen by patients and/or by healthcare professionals, but they are not specific for evaluating package leaflets.^8^^,^^9^

In contrast, other tools such as the Consumer Information Rating Form were specifically developed to evaluate consumers’ perceptions of the comprehensibility, utility and design quality of written information relating to medicines, such as package leaflets. This tool is composed of the following evaluations: perceptions of comprehensibility (five items), intended future use, perceived usefulness of information (eight items) and design quality (seven items). Only the perceived comprehensibility and usefulness of the information are assessed on a five-point scale, from 1 (very hard) to 5 (very easy).^6^

Tools using Likert scales for these purposes have also not been reported for Portuguese package leaflets.^4^ Pires et al. found that sociodemographic characteristics explained Portuguese users’ opinions of package leaflets: lower socioeconomic status or higher frequency of taking medicines positively influenced participants’ overall opinion and/or perception regarding package leaflets.^5^ Worryingly, previous studies detected that package leaflets of authorized medicines were too complex, and were difficult to understand and use, thus confirming that even approved package leaflets need to be optimized.^5^^,^^10^^,^^11^^,^^12^

## OBJECTIVE

The aim of this study was to validate a questionnaire (LiS-RPL) that had been designed to evaluate subjects’ satisfaction with the readability of package leaflets for medicines*.*

## METHODS

### Setting and ethical approval

This study formed part of a larger research project that had previously been communicated to INFARMED, I.P. (the Portuguese medicines agency), and to the National Data Protection Commission (CNPD) of Portugal.^11^ Subjects were informed of the nature and general goals of the study and voluntarily agreed to answer the questionnaires. They were also informed that they could leave the study at any moment. Furthermore, this study did not involve administration of medicines or other substances to humans or animals.

It was conducted within the development of a PhD thesis on Pharmacy (Socio-Pharmacy; School of Pharmacy of the University of Lisbon), which was announced in official statement no. 4719/2016 in the Official Gazette of the Portuguese Republic (https://dre.pt/application/file/a/74059402).

### Participants

Overall, 503 participants were selected according to convenience, in two Portuguese regions (49.3% of the participants were living in urban areas and 50.7% in rural areas), during 2014. Public and private schools, municipalities, the army or other institutions with a considerable number of collaborators/employees were contacted by email or telephone. In case of acceptance, a day was defined for administering the questionnaires. Around half of the participants were male (53.1%), and the participants’ education level was stratified into three groups: 32% had had 1-6 years of schooling; 37%, 7-12 years of schooling; and 31%, more than 12 years of schooling (or higher education in Portugal). More details on the study design can be found in previous studies.^5^^,^^10^^,^^11^

### Instrument

The tool for evaluating the subjects’ perceptions of or satisfaction with the readability of package leaflets (LiS-RPL) was developed based on the criteria of the European Guideline on the Readability of Package Leaflets, and on the Consumer Information Rating Form, which is used to evaluate information for consumers, such as package leaflets.^1^^,^^6^

Among the items considered in the Consumer Information Rating Form, those referring to intended future use and perceived usefulness of information were not included in our tool, because these issues are not specifically described in the European Guideline on the Readability of Package Leaflets.^1^ Furthermore, these issues may be considered to be more subjective and dependent on patients’ previous knowledge of their health condition.

Our questionnaire was composed of 14 items, since the European Guideline recommends the development of questionnaires comprising 12 to 15 questions in order not to tire out the participants.

The LiS-RPL analysis is presented in Tables 1 and 2, and the Portuguese version in Table 3. Each item was classified using a Likert scale of 1-5 to rate the level of satisfaction or perception according to the following labels: 1 = poor; 2 = not very satisfied; 3 = no opinion; 4 = satisfied; and 5 = good (Table 3).^3^^,^^13^ A similar tool was pretested in a previous study (n = 63 participants), in which the opinions of physicians, pharmacists and potential users of medicines regarding the readability of the package insert of an over-the-counter medicine were collected.^3^

**Table 1.** Descriptive statistics for the items that were designed to evaluate subjects’ satisfaction with the readability of package leaflets for medicines (LiS-RPL) (n = 469)

Items of LiS-RPL*	Mean	SD	Skewness	Kurtosis
Item 1 - Font size	3.47	1.44	-0.55	-1.14
Item 2 - Font type	4.13	1.11	-1.38	1.13
Item 3 - Layout of the titles of the sections	4.07	1.02	-1.11	0.62
Item 4 - Color of the text	4.30	0.95	-1.53	2.20
Item 5 - Line spacing	3.85	1.23	-0.86	-0.45
Item 6 - Use of the en-dash throughout the text	4.14	0.98	-1.22	1.21
Item 7 - Clarity of the text	3.84	1.16	-0.91	-0.90
Item 8 - Length of the sentences	4.03	1.08	-1.05	0.33
Item 9 - Number of sentences in each paragraph	4.02	1.13	-1.07	0.30
Item 10 - Description of possible side effects	3.90	1.10	-0.87	-0.17
Item 11 - Comprehensibility of medical terms	3.66	1.17	-0.56	-0.74
Item 12 - Clarity of the instructions for the user	3.96	1.07	-0.99	0.24
Item 13 - Use of abbreviations throughout the text	3.45	1.18	-0.31	-0.78
Item 14 - Repetition of the brand name of the medicine throughout the text	4.01	1.01	-0.98	0.46

SD = standard deviation.

*Each item was classified using a Likert scale of 1-5, to assess the level of satisfaction/perception: 1 = poor; 2 = not very satisfied; 3 = no opinion; 4 = satisfied; and 5 = good.

**Table 2.** Pattern matrix from exploratory factor analysis on the questionnaire that was designed to evaluate subjects’ satisfaction with the readability of package leaflets for medicines (LiS-RPL) (n = 469)

Items of LiS-RPL*	Factor 1 Clarity and comprehension of text	Factor 2 Format	h^2^
Item 12 - Clarity of the instructions for the user	1.01		0.74
Item 11 - Comprehensibility of medical terms	0.90		0.62
Item 7 - Clarity of the text	0.81		0.61
Item 10 - Description of possible side effects	0.76		0.66
Item 2 - Font type		1.10	0.67
Item 5 - Line spacing		0.86	0.61
Item 4 - Color of the text		0.80	0.61
Item 1 - Font size		0.78	0.34
Item 3 - Layout of the title of the sections		0.70	0.55
Item 9 - Number of sentences in each paragraph		0.57	0.68
Item 8 - Length of the sentences		0.57	0.62
Item 6 - Use of the en-dash throughout the text		0.52	0.55
Eigenvalue	6.74	1.29	-
Total variance explained (%)	56.10	10.76	-

*Each item was classified using a Likert scale of 1-5, to assess the level of satisfaction/perception: 1 = poor; 2 = not very satisfied; 3 = no opinion; 4 = satisfied; and 5 = good.

**Table 3.** Responda ao questionário assinalando com um número de 1 a 5 a opção que melhor se adequa à sua opinião:

		1 - Mau 2 - Pouco satisfeito 3 - Sem opinião 4 - Satisfeito 5 - Bom
	Como classifica:	Responda de 1 a 5
1.	O tamanho da letra	
2.	O tipo de letra	
3.	A apresentação dos títulos	
4.	A cor do texto	
5.	Os espaços entre as linhas	
6.	A utilização de listas de informações no texto	
7.	A simplicidade da linguagem	
8.	O tamanho das frases	
9.	O tamanho dos parágrafos	
10.	A forma como é dada a informação sobre os efeitos secundários	
11.	A simplicidade dos termos médicos	
12.	A forma de dar instruções ao doente	
13.	A repetição do nome do medicamento ao longo do folheto	
14.	A sua satisfação geral com a forma como a informação é dada	

In the present study, the labels of satisfaction or perception were redesigned based on the supposition that the use of two labels of quality (poor and good), two labels of satisfaction (not very satisfied and satisfied) plus a neutral point (no opinion) might contribute towards improving participants’ understanding. Also, LiS-RPL was pretested on the first 50 study participants. All of the participants used this tool correctly, i.e. no usability issues were identified.

### Package leaflets selected for evaluation

The package leaflets that were tested were randomized using the MS Excel software function from a large database used in a previous study (with approximately 500 Portuguese package leaflets).^14^ A total of 12 randomized package leaflets were tested: six package leaflets from over-the-counter medicines (three package leaflets comprising more than 1500 words and another three with less than 1500 words) and six package leaflets from prescription medicines (also with either more than or less than 1500 words). The cutoff of 1500 words was defined because previous studies had concluded that package leaflets with fewer than 1500 words tended to be easily read and understood.

These package leaflets (grouped as described above) were distributed equally among three groups of participants that were defined according to their numbers of years of schooling (1-6, 7-12 or > 12).

More details on the selection and type of package leaflets can be consulted in Table 4 and in the study by Pires et al.^5^^,^^10^^,^^11^ All the package leaflets that were tested were organized in accordance with the template of the European Medicine Agency and were composed of the following sections: 1. What X is and what it is used for; 2. What you need to know before you <take> <use> X; 3. How to <take> <use> X; 4. Possible side effects; 5. How to store X; and 6. Contents of the pack.^15^

**Table 4.** Selection of the leaflets among participants according to education level groups and the features of the package leaflets tested.

Education level	Features of package leaflets - part A						
	Active substances	Therapeutic groups	Pharmaceutical presentations	Type of dispensing	Length (words)**	Font size	Font type
0-6 years of schooling	Gelatin (78 mg or 5532 mg) + glycerol (6.5 g)	Drugs altering gut motility	Rectal gel	Over-the-counter	≤ 1,500 (966 words)	7	Arial
	Minoxidil (50 mg/ml)	Topical products for hair loss	Solution	Over-the-counter	> 1,500 (2,220 words)	8	Arial
	Cefatrizine (50 mg/ml)	Antibacterial drugs	Oral suspension powder	Prescription medicine	≤ 1,500 (1,465 words)	8	Arial
	Ipratropium bromide (0.52 mg/2.5 ml) + salbutamol (3 mg/2.5 ml)	Antiasthmatics	Aerosol	Prescription medicine	> 1,500 (1,683 words)	8	Arial
7-12 years of schooling	Povidone-iodine (100 mg/ml)*	Vaginal disinfectant	Solution	Over-the-counter	≤ 1,500 (1,075 words)	6	Arial
	Oxymetazoline (0.5 mg/ml)	Nasal decongestants	Nasal spray	Over-the-counter	> 1,500 (1,714 words)	8	Arial
	Ofloxacin (3 mg/ml)	Topical antibacterials	Ophthalmic drops	Prescription medicine	≤ 1,500 (1,345 words)	8	Other sans serif
	Clomipramine (10/25/75 mg)	Antidepressants	Tablets	Prescription medicine	> 1,500 (2,699 words)	10	Arial
> 12 years of schooling	Choline salicylate (87 mg/g)	Anti-ulcerants	Oral gel	Over-the-counter	≤ 1,500 (924 words)	6	Arial
	Acetylsalicylic acid (400 mg) + ascorbic acid (240 mg)	Analgesic and antipyretics	Effervescent tablets	Over-the-counter	> 1,500 (2,346 words)	7	Arial
	Dexamethasone (1 mg/ml) + neomycin (10 mg/ml) + polymyxin B (10000 IU/ml)	Topical corticosteroids	Optical drops	Prescription medicine	≤ 1,500 (1,056 words)	8	Arial
	Methylprednisolone (40 mg/ml) + lidocaine (10 mg/ml)	Corticosteroids	Parenteral injection	Prescription medicine	> 1,500 (3,487 words)	7	Arial

*Only female participants received these questionnaires (A and B); ** Package leaflets with ≤ 1,500 words (average = 1,138.5 words, standard deviation = 198.3) and package leaflets with > 1,500 words (average = 2,358.2; standard deviation = 616.5).

**Table:** 

Education level	Features of package leaflets - part B								
	Active substances	Layout of the title	Color	Line Spacing	En-dash‡	Length of sentences (average no. of words)	No. of paragraphs	Abbreviations	Repetition of brand names
0-6 years of schooling	Gelatin + glycerol	Capitalized	Black	≤ 1 pt	1	13.8	15	5	1 ‡‡
	Minoxidil	Capitalized	Black	> 1 pt	6	14.9	54	12	1
	Cefatrizine	Capitalized	Black	> 1 pt	5	14.2	39	12	1
	Ipratropium bromide	Capitalized	Black	> 1 pt	8	17.2	38	4	1
7-12 years of schooling	Povidone-iodine	Not capitalized	Black	> 1 pt	1	12.4	29	1	1
	Oxymetazoline	Not capitalized	Yes	> 1 pt	6	13.5	57	15	0 ‡
	Ofloxacin	Capitalized	Black	> 1 pt	5	14.5	30	1	1
	Clomipramine	Capitalized	Black	> 1 pt	4	17.6	28	16	1
> 12 years of schooling	Choline salicylate	Capitalized	Black	> 1 pt	1	14.7	6	3	1
	Acetylsalicylic acid + ascorbic acid	Capitalized	Yes	> 1 pt	8	14.8	31	16	0
	Dexamethasone + neomycin	Not capitalized	Black	> 1 pt	3	14.7	25	5	1
	Methylprednisolone + lidocaine	Capitalized	Black	> 1 pt	4	16.5	45	5	0

‡ = Number of groups of items with en-dash; ‡‡ = The name of medicine appears at least once per more than 50 words.

### Procedure

A day was previously scheduled in all institutions that accepted the invitation to participate in the present study: participants were invited to be present, but they were free to quit. A colored printed version of the package leaflets and the LiS-RPL were distributed. The participants were told not to consult the package leaflet before all the instructions had been issued. A researcher explained aloud how to use LiS-RPL, and all participants were invited to clarify any queries that they might have regarding how to use the scale, before starting to complete the questionnaire.

The participants were required to classify their satisfaction with or perception of aspects of the package leaflet that was being tested, with regard to clarity, simplicity and comprehensibility of the text, and any typographic or printing issues. The LiS-RPL was self-administered.^3^ All participants completed the task in less than 15 minutes.

### Data analysis

#### Descriptive statistics

For the present study, questionnaires in which any of the values on any of the scale items had not been filled out were excluded. Thus, the final sample comprised 469 completed forms. The first objective was to describe the central trend, dispersion and distribution of the ratings of all the items. Multivariate tests for skewness and kurtosis, as proposed by Mardia, were also examined. Significant results in the Mardia test supported our decision to use polychoric correlations.^16^

#### Dimensionality

To examine dimensionality (which was our second objective), identification and fine-tuning of the instrument factor structure was conducted through an exploratory factor analysis. The number of factors was decided upon based on the results from the minimum average partial method in conjunction with the results from the parallel analysis using minimum rank factor analysis. This was based on random permutation of the sample data and comparison with the percentage of common variance that was extracted via minimum rank factor analysis.^17^^-^^18^

#### Number of factors to retain

An in-depth analysis based on the percentage of explained variance and on root-mean-square residuals was performed to select the number of factors to retain.^19^ Root-mean-square residuals summarize the residual covariance matrix and the model fit, such that lower values represent a better model fit. Since the two-factor solution provided a higher percentage of explained variance and lower root-mean-square residuals, compared with the single-factor solution, the exploratory factor analysis was forced into two factors.

An exploratory factor analysis with a unweighted least-squares extraction method using a polychoric matrix was performed. The unweighted least-squares extraction method was chosen because it produces inter-factor correlation estimates of greater accuracy.^20^ The promin oblique rotation method was applied to gain better solutions in the ordinal dataset, thereby allowing factors to be oblique so that factor simplicity could be maximized.

#### Kaiser-Meyer-Olkin index and Bartlett’s sphericity test

The adequacy of the matrix for exploratory factor analysis was examined by assessing the determinant, the Kaiser-Meyer-Olkin index and Bartlett’s sphericity test. Only factor loadings ≥ 0.40 were considered substantive.^21^ Items with low communalities (*h* ^*2*^ < 0.30) or cross-loading items (item loading at 0.40 or higher in two or more factors) were eliminated. Whenever low factor loadings, low communalities or cross-loadings were found, any such items were removed and exploratory factor analysis was performed again until a stable structural solution was found.^19^^,^^22^

#### Exploratory bifactor analysis

An exploratory bifactor analysis with the Schmid-Leiman solution was performed to examine the possible existence of a broad second-order factor that would directly influence the observed variables.^23^ Performing this exploratory bifactor analysis allowed us to determine whether a measurement could be treated as a single factor, or whether it would be best represented as separate but related factors.^24^

In a bifactor model, an overall factor accounts for relationships between individual items (akin to a single-factor model) and is labelled as a general factor.^25^ Additionally, an IBM-SPSS syntax written by Wolf and Preising was used to calculate the total extracted variance accounted for by the general factor and first-order factors.^26^

#### Bentler’s Simplicity Index and Loading Simplicity Index

The factor simplicity was assessed through Bentler’s Simplicity Index and Loading Simplicity Index, in which greater values represent simpler and more interpretable solutions.^27^^,^^28^

#### Reliability of the instrument

Lastly, the reliability of the instrument was assessed using tests of internal consistency and homogeneity for each of the subscales and the overall score. Cronbach’s alpha, ordinal alpha and inter-item correlation coefficients were used for the subscales. Ordinal alpha was computed manually, since it has been shown to estimate reliability more accurately than Cronbach’s alpha for ordinal response scales.^29^^-^^30^

The overall scale score was assessed using McDonald’s total omega coefficient (ωt), which represents the proportion of total common variance in the instrument.^31^ Lastly, McDonald’s hierarchical omega coefficient (ωh) was computed manually for the bifactor structure, following the recommendations of Widhiarso and Ravand (2014).^31^^-^^32^ McDonald’s hierarchical omega coefficient can be seen as an estimate of the general factor saturation of an instrument, thus enabling examination of the extent to which a overall score is interpretable as a measurement of a single common factor.^33^^,^^34^^,^^35^

Cronbach’s alpha, ordinal alpha and McDonald’s omega (ωt and ωh) with values higher than 0.7 and mean item correlation between 0.15 and 0.50 were regarded as acceptable.^30^^,^^31^ Correlation coefficients were interpreted in accordance with the criteria described by Cohen.^33^

#### Statistical software

Descriptive and reliability analyses were carried out using the IBM-SPSS statistics software, version 25.0. Exploratory factor analysis and exploratory bifactor analysis were both conducted via the Factor software, version 10.8.^34^

## RESULTS

### Descriptive statistics

Descriptive statistics for each item are presented in Table 1. Item 4 (color of the text) showed the highest values for the mean and dispersion. Skewness and kurtosis values were within ranges that were adequate for univariate normal distribution. However, Mardia’s test showed the presence of excessive multivariate kurtosis (*K*_*2*_ = 331.12; P < 0.001).^16^

### Dimensionality examination

The minimum average partial method and minimum rank factor analysis indicated a two-factor structure and a single-factor structure, respectively. The matrix determinant was > 0.001 and the Kaiser-Meyer-Olkin value was 0.90, which confirmed the adequacy of the sample. The significance of the result from Bartlett’s sphericity test, i.e. χ^2^ (98) = 2851.70; P < 0.001, meant that the polychoric correlations between the items were large enough to conduct exploratory factor analysis.

### Number of factors to retain

The exploratory factor analysis on the 14 items showed a two-factor structure that explained 60.0% of the variance. However, item 13 (use of abbreviations throughout the text) and item 14 (repetition of the brand name of the medicine throughout the text) were removed due to low communalities (< 0.3) and factor loadings (< 0.4), and the exploratory factor analysis was performed again. The pattern matrix from the exploratory factor analysis on 12 items and the communalities are shown in Table 2.

Together, the two factors explained 69.11% of the variance. Factor 1 (clarity and comprehension of text) comprised four items and accounted for 56.10% of the variance, while factor 2 (format) consisted of the remaining eight items and accounted for 10.76% of the variance.

No cross-loadings were found in the pattern matrix, and all significant item loadings were greater than 0.4. Similarly, the factor simplicity indices were also adequate, such that Bentler’s Simplicity Index was 0.98 (percentile 99) and the Loading Simplicity Index was 0.48 (percentile 98).^27^ The two factors were strongly correlated with each other (r = 0.80), thus supporting non-orthogonality.

### Exploratory bifactor analysis with Schmid-Leiman solution

According to the Schmid-Leiman solution, all items seemed to represent the general factor because they showed loadings above 0.50.^23^ The range of factor loadings was between 0.51 (item 1: font size) and 0.79 (item 9: number of sentences in each paragraph).

The loadings of the two first-order factors on the second-order factor were 0.84 and 0.95 for factor 1 (clarity and comprehension of text) and factor 2 (format), respectively. The results from the Schmid-Leiman solution for the present instrument are shown in Table 5.

**Table 5.** Results from Schmid-Leiman solution produced through a questionnaire that was designed to evaluate subjects’ satisfaction with the readability of package leaflets for medicines (LiS-RPL), in a sample of 469 participants

Item	F1 First-order factor	F2 First-order factor	G Second-order factor
Item 1 - Font size	-0.15	0.24	0.51
Item 2 - Font type	-0.19	0.34	0.72
Item 3 - Layout of the title of the sections	0.03	0.22	0.71
Item 4 - Color of the text	-0.15	0.25	0.74
Item 5 - Line spacing	-0.06	0.27	0.73
Item 6 - Use of the en-dash throughout the text	0.14	0.16	0.71
Item 7 - Clarity of the text	0.43	-0.01	0.65
Item 8 - Length of the sentences	0.14	0.18	0.76
Item 9 - Number of sentences in each paragraph	0.16	0.18	0.79
Item 10 - Description of possible side effects	0.41	0.02	0.70
Item 11 - Comprehensibility of medical terms	0.48	-0.04	0.62
Item 12 - Clarity of the instructions for the user	0.55	-0.07	0.66
Variance explained (%)	76.40	18.10	5.50

The general factor accounted for 76.4% of the extracted variance, a proportion that was evidently above the range that would be considered to be indicative of the presence of a general factor (40-50%), whereas the two first-order factors explained 18.10% (factor 1: clarity and comprehension of text) and 5.50% (factor 2: format) of the variance.^22^

### Hierarchical solution

The hierarchical solution according to two first-order factors and one second-order factor (G) exhibited good simplicity indices, such that Bentler’s Simplicity Index was 0.99 and the Loading Simplicity Index was 0.48 (percentile 99).^27^

### Reliability

All the internal consistency indexes indicated good reliability of measurement (Table 6). The mean inter-item correlation of factor 1 was only slightly above 0.5, which can be considered satisfactory. The overall scale score showed a mean inter-item correlation of 0.43, indicating that the items forming the scale were homogenous.^31^^,^^35^

**Table 6.** Internal consistency and homogeneity measurements for the questionnaire that was designed to evaluate subjects’ satisfaction with the readability of package leaflets for medicines (LiS-RPL)

	Cronbach’s alpha	Ordinal alpha	Mean inter-item correlation	McDonald’s total omega	McDonald’s hierarchical omega
Factor 1 - Clarity and comprehension of text	0.85	0.92	0.58	-	
Factor 2 - Format	0.86	0.90	0.45	-	
Overall scale score	-	-	0.43	0.93	0.85

## DISCUSSION

### Participants

Overall, 469 (93.2%) out of 503 (100%) sets of results from LiS-RPL were included (Tables 1 and 2), which is in line with the response rates in other studies involving Likert scales, i.e. response rates of 84%-91%.^7^ Since the participants were selected according to convenience in only two regions of Portugal, the data collected is possibly not representative of the entire Portuguese population.

### Tool evaluated (LiS-RPL)

#### Items removed

Two items were removed: item 13 (use of abbreviations throughout the text) and item 14 (repetition of the brand name of the medicine throughout the text), since they did not contribute significantly to the construct of the LiS-RPL. Interestingly, like the remaining purposed items, numbers 13 and 14 were based on the recommendations of the European Guideline on the Readability of Package Leaflets: abbreviations and acronyms should not usually be used, and in general a reference to “your medicine, this medicine, etc.” is considered more suitable than repeating the name of the product, respectively.^1^

These items (13 and 14) were removed because their contribution was not statistically significant. The reasons for this may have been the following: readers are accustomed to consulting texts containing abbreviations within their daily routine, such as in the texts of newspapers or the internet; and, furthermore, the names of medicines are heterogeneously distributed in package leaflets.

#### Items selected

The scale was composed of 12 items that presented a stable structure (high communalities, high factor loadings and explanation of almost 70% of the variance). The variance explained was above the acceptable level (50%).

It was possible to identify two dimensions in factor 1 (clarity and comprehension of text) and in factor 2 (format). Factor 1 was composed of four items: clarity of the patients’ instructions (item 12); comprehensibility of the medical terms (item 11); clarity of the text (item 7); and description of the possible side effects (item 10). Factor 2 (format) was composed of eight items: font type (item 2); line spacing (item 5); color of the text (item 4); font size (item 1); layout of the title of the sections (item 3); number of sentences in each paragraph (item 9); length of the sentences (item 8); and use of the en-dash throughout the text (item 6).

In addition to being stable and valid, this structure seemed to be coherent, since factors 1 and 2 contributed to the same types of issues: one relating to content and the other to form. Out of the 14 items thus selected, the color of the text was the most variable factor in participants’ responses. This may have been related to the diversity of social and educational factors in our sample.

Regarding the results from the bifactor analysis, we cannot rule out the possibility that the scale, as a whole, might be considered unidimensional. McDonald’s omega coefficient indicated that this total score was very strongly correlated with the hypothetical domain in which the items formed a subset, thus supporting the computation of the total score for the scale.^31^ This scenario may be explained by the fact that the clarity and comprehension (Factor 1) of the text and format (Factor 2) issues are strongly interrelated with regard to the readability and intelligibility of package leaflets, since both dimensions contribute towards readers’ comprehension.^4^

### Acceptability rate and recommendations

LiS-RPL is a reliable and validated tool for evaluating participants’ satisfaction with or perception of package leaflets, for the European Portuguese-speaking population. LiS-RPL addresses two dimensions relating to package leaflets: the clarity and comprehension of the text and format issues.

It seems that government policy and/or health promotion interventions should include specific measures to ensure that package leaflets are truly comprehensible and usable, such as application of validated tools. These matters are scarcely represented in regulations.

### Limitations

The Likert scale used did not follow the usual label pattern, i.e. very satisfied, fairly satisfied, neutral, not very satisfied or not at all satisfied.^3^^,^^36^ This may have introduced the possibility of linguistic constraints, such as (i) the differences between the labels *satisfeito* (satisfied) and *bom* (good) may not have been clear to all participants; and (ii) comparison of the labels *pouco satisfeito* (not very satisfied) versus *bastante satisfeito* (very satisfied) may be considered more suitable than *pouco satisfeito* (not very satisfied) versus *satisfeito* (satisfied). Nevertheless, the ordinal nature of the scale from a completely negative to a completely positive response can be assumed to have been preserved. In this regard, additional linguistic and statistical evaluations are recommended in future studies.^36^

## CONCLUSION

LiS-RPL is a reliable and validated tool for evaluating participants’ satisfaction with or perception of package leaflets for the European Portuguese-speaking population. LiS-RPL addresses two dimensions of package leaflets: the clarity and comprehension of the text and format issues.
